# The Prognostic Value of Radiomics Features Extracted From Computed Tomography in Patients With Localized Clear Cell Renal Cell Carcinoma After Nephrectomy

**DOI:** 10.3389/fonc.2021.591502

**Published:** 2021-03-05

**Authors:** Xin Tang, Tong Pang, Wei-feng Yan, Wen-lei Qian, You-ling Gong, Zhi-gang Yang

**Affiliations:** ^1^ Department of Radiology, West China Hospital, Sichuan University, Chengdu, China; ^2^ Department of Thoracic Oncology and State Key Laboratory of Biotherapy, Cancer Center, West China Hospital, Sichuan University, Chengdu, China

**Keywords:** radiomics, computed tomography, clear cell renal cell carcinoma, prognosis, predictive model

## Abstract

**Background and purpose:**

Radiomics is an emerging field of quantitative imaging. The prognostic value of radiomics analysis in patients with localized clear cell renal cell carcinoma (ccRCC) after nephrectomy remains unknown.

**Methods:**

Computed tomography images of 167 eligible cases were obtained from the Cancer Imaging Archive database. Radiomics features were extracted from the region of interest contoured manually for each patient. Hierarchical clustering was performed to divide patients into distinct groups. Prognostic assessments were performed by Kaplan–Meier curves, COX regression, and least absolute shrinkage and selection operator COX regression. Besides, transcriptome mRNA data were also included in the prognostic analyses. Endpoints were overall survival (OS) and disease-free survival (DFS). Concordance index (C-index), decision curve analysis and calibration curves with 1,000 bootstrapping replications were used for model’s validation.

**Results:**

Hierarchical clustering groups from nephrographic features and mRNA can divide patients into different prognostic groups while clustering groups from corticomedullary or unenhanced phase couldn’t distinguish patients’ prognosis. In multivariate analyses, 11 OS-predicting and eight DFS-predicting features were identified in nephrographic phase. Similarly, seven OS-predictors and seven DFS-predictors were confirmed in mRNA data. In contrast, limited prognostic features were found in corticomedullary (two OS-predictor and two DFS-predictors) and unenhanced phase (one OS-predictors and two DFS-predictors). Prognostic models combining both nephrographic features and mRNA showed improved C-index than any model alone (C-index: 0.927 and 0.879 for OS- and DFS-predicting, respectively). In addition, decision curves and calibration curves also revealed the great performance of the novel models.

**Conclusion:**

We firstly investigated the prognostic significance of preoperative radiomics signatures in ccRCC patients. Radiomics features obtained from nephrographic phase had stronger predictive ability than features from corticomedullary or unenhanced phase. Multi-omics models combining radiomics and transcriptome data could further increase the predictive accuracy.

## Introduction

Renal cell carcinoma (RCC) is the third most prevalent malignancy of urological tumors (1). It is estimated that 80–90% RCCs belong to clear cell RCC (ccRCC) (2). For patients with localized ccRCC, nephrectomy remains to be the standard treatment. However, even after surgery, disease progression can still occur in many patients. Besides, due to the tumor heterogeneity, the prognosis of ccRCC varies from cases to cases. Precise prognostic prediction for ccRCC patients is not only important for patients’ counseling but also essential for clinicians making personalized therapeutic decision.

Computed tomography (CT) scan plays a critical role in RCC diagnosis and is also one of the routine examinations for post-treatment disease assessment. Yet, in clinic, the interpretation of CT images relies largely on the experience of radiologists, thus, lacking quantitative information and occasionally showing inter-observer inconsistency. In contrast, the recent emerging technique of radiomics texture analysis provides more objective and quantitative details for medical images. Thereby, it has great potential in assessing the heterogeneity of tumors. Many studies reported that radiomic analysis harbors promising ability in predicting oncologic characteristics such as malignant lesion, pathological type, tumor stage and *etc.* as well as non-oncologic disease (3–6). Furthermore, radiomics features were also associated with treatment response and prognosis in several tumors (7–9).

In kidney-related disease, radiomics analysis showed wide applications. CT texture analysis was capable of distinguishing benign and malignant renal masses and predicting the Fuhrman nuclear grade of RCC accurately (10–12). Besides, CT radiomics features could also assist in differentiating kidney stones from phleboliths (6). In addition, texture analysis was able to facilitate the assessment of renal allograft function after kidney transplantation (13). However, till now, little is known as to whether radiomics features extracted from different CT phases have prognostic value in predicting the survival outcomes of ccRCC patients after nephrectomy.

Apart from radiomics analyses, gene expression profiling also showed marked significance in prognostic evaluation in many cancers. Therefore, the aim of the current study is to explore the role of radiomics features extracted from CT images in predicting the postoperative prognosis of patients with localized ccRCC. In addition, we investigated if the combination of both radiomics features and transcriptome mRNA would further increase the predictive accuracy.

## Material and Methods

### Patients and Data Acquisition

**Figure 1:**
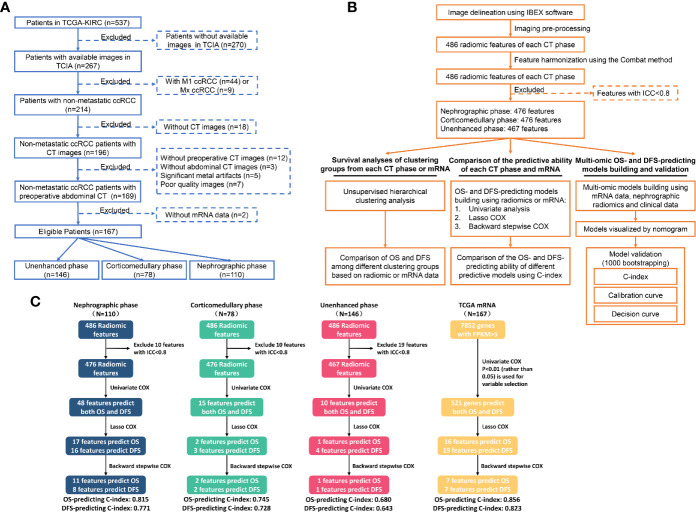
Flowchart showing the inclusion criteria **(A)**, the detailed analytic strategy **(B)** and the results of the prognostic analyses **(C)**. TCGA, The Cancer Genome Atlas; TCIA, The Cancer Imaging Archive; ccRCC, Clear cell renal cell carcinoma; CT, Computed tomography; OS, Overall survival; DFS, Disease-free survival.

No ethical approval or informed consent was needed for this study because all data we used were from public databases. Clinicopathological data and CT images were obtained from the Cancer Imaging Archive (TCIA) database (http://www.cancerimagingarchive.net/) (14). TCIA is the U.S. National Cancer Institute’s image repository supporting cancer research which contains millions of public oncology images. While transcriptome mRNA data were obtained from the Cancer Genome Atlas (TCGA) database (http://cancergenome.nih.gov) (15). TCGA is an openly web-accessible database collecting molecular information of 33 different cancer types. The inclusion criteria are shown in **Figure 1A** which mainly include: 1) patients with available CT images of good quality; 2) patients with M0 ccRCC, and 3) patients with accessible mRNA data. Eventually, 167 out of 537 patients from the TCGA-KIRC cohort were eligible.

### CT Imaging

All abdominal CT images of included ccRCC patients were acquired before nephrectomy. The imaging data were collected from seven institutions and three different manufactures (General Electric (GE), Siemens and Philips Medical Systems). The acquisition parameters of CT were as follows: slice thickness, 1–5mm; tube voltage, 120–140 kV; tube current, 160–618 mA; display field of view, 278–628; matrix, 512 × 512; and pixel size, 0.542 × 0.542 mm^2^ to 0.976 × 0.976 mm^2^. The image format of DICOM was used in this study. Radiomics modeling details according to the Imaging Biomarker Standardization Initiative (IBSI) guidelines are shown in **Table S1**.

### Data Pre-Processing

Pre-processing steps were performed in all images to reduce the potential influences of protocol variability from various institutions and CT scanners. Specifically, image pre-processing and features pre-processing were carried out before data analysis. Image pre-processing includes voxel resampling and gray-level discretization. The cubic B-spline interpolation method was employed for voxel resampling (resampled pixel spacing = 1 × 1 mm^2^) (16). The fixed bin size (FBS) discretization method (bin widths = 25 HU) was used for gray-level discretization (17). As for feature pre-processing, Combat algorithm was conducted for feature harmonization (https://github.com/Jfortin1/ComBatHarmonization) (18, 19). Besides, Z-score transformation was used for data normalization.

### Feature Extraction

CT images were acquired before surgery. On the axial image slice, the regions of interest (ROI) of the tumor with the largest cross-sectional area were selected and contoured manually using the open-source software Imaging Biomarker Explorer (IBEX) by two radiologists independently (20). In order not to cover the adjacent normal renal tissue, the radiologists determine the tumor margin with the guide of available contrast-enhanced CT during segmentation of unenhanced CT. Finally, 486 radiomics features were extracted from nephrographic, corticomedullary and unenhanced phase, including gray-level co-occurrence matrix (GLCM), gray-level run length matrix (GLRLM), shape, gradient orient histogram (GOH), neighbor intensity difference (NID), and intensity histogram. Most definitions of IBEX feature are compliant with Image Biomarkers Standardization Initiative (IBSI) (21).

### Prognostic Analyses and Radiotranscriptomics Models’ Building

All radiomic features were standardized using the Z-score transformation. The agreement upon radiomics features between the two radiologists were examined by inter-rater interclass correlation coefficient (ICC). Only features with inter-rater ICC >0.8 were further analyzed. As for transcriptome data, only genes with median FPKM mRNA value >0.5 were included.

The endpoints or predictive objects of this study were overall survival (OS) and disease-free survival (DFS). OS was the period from the initial diagnosis of ccRCC to death, while DFS was the duration from the initial diagnosis to the date of cancer progression or death.

The prognostic analyses consisted of three parts. In the first part, we applied unsupervised hierarchical clustering analyses using radiomics features from different CT phases and mRNA data, which classified patients into different clustering groups. Hierarchical clustering was conducted based on Euclidean distance. Then, to explore the overall value of radiomic features from each CT phase and the mRNA data in predicting patients’ prognosis, OS and DFS were compared within the clustering groups from each CT phase or mRNA data.

Secondly, we test and compare the predictive ability of radiomic features extracted from each CT phase and transcriptome mRNA in prognostic assessment, which was carried out as follows: firstly, univariate COX regression was performed to identify potential prognosticators from radiomic features and mRNA data. Then, radiomic features with p<0.05 and genes with p<0.01 in univariate analyses were further tested by the least absolute shrinkage and selection operator (LASSO) COX regression methods. Finally, backward stepwise COX was used to simplify the predictive models. The predictive accuracy of these models based on each CT phase and transcriptome data were compared by the concordance index (C-index).

The third part of prognostic evaluation was to build prognostic models by integrating radiotranscriptomics data together aiming at developing more accurate prognostic assessment tools. In this part, two prognostic models respectively predicting OS and DFS were developed using the 110 cases with available nephrographic phase data and mRNA data and were visualized by nomograms. Additionally, the models were tested by 1,000 bootstrapping replications. In this process, three distinct aspects of the final models were evaluated, *i.e.* discrimination ability examined by C-index; clinical benefit assessed by decision curve analyses (DCA) and consistency between observation and prediction by calibration curve.

### Statistical Analyses

Radiomics features were extracted from CT imagines using the IBEX software. Statistical analyses were performed using R software (V 3.6.2). R packages used in this study include “pheatmap”, “survival” and “glmnet”. All tests were two-sided. A *p* value <0.05 was considered significant for all the tests except in univariate COX regression for mRNA data, a *p* value <0.01 was defined as significant.

## Results

### Patients’ Characteristics

According to our inclusion criteria, 167 eligible patients with M0 ccRCC were included (**Figure 1A**). Available images of nephrographic, corticomedullary and unenhanced phase were obtained from 110 (65.9%), 78 (46.7%) and 146 (87.4%) cases, respectively, while transcriptome mRNA data was accessible for all patients. The baseline characteristics of all cases are summarized in **Table 1**. The median follow-up time using the reverse Kaplan–Meier method was 50.0 Mo. In total, death and disease progression occurred in 34/167 (20.4%) and 46/167 (27.5%) cases, respectively. The median OS was not reached, while the median DFS was 118.8-Mo (95%CI: 84.6–152.9 Mo).

**Table 1:** Baseline characteristics of the total patients included in the current study.

Age	
**Median (IQR)**	59.0 (51.0-70.0)
**Diagnosis Year**	
** 2000-2005**	75 (44.9%)
** 2006-2010**	92 (55.1%)
**Sex**	
** Male**	54 (32.3%)
** Female**	113 (67.7%)
**Laterality**	
** Left**	74 (44.3%)
** Right**	93 (55.7%)
**T stage**	
** T1-2**	118 (70.7%)
** T3-4**	49 (29.3%)
**N stage**	
** N0**	74 (44.3%)
** N1**	2 (1.2%)
** Nx**	91 (54.5%)
**Fuhrman grade**	
** Grade I-II**	75 (44.9%)
** Grade III-IV**	92 (55.1%)
**Available CT phase**	
** Nephrographic phase**	110 (65.9%)
** Corticomedullary phase**	78 (46.7%)
** Unenhanced phase**	146 (87.4%)

IQR, interquartile range.

### Unsupervised Hierarchical Clustering Analyses

**Figure 2:**
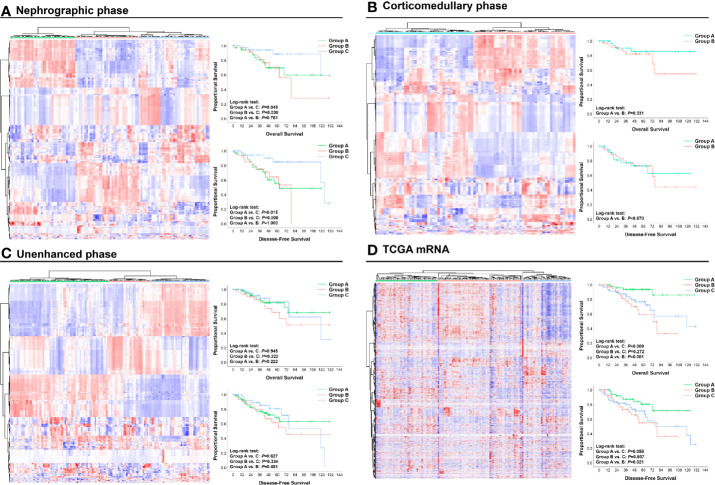
Unsupervised hierarchical clustering analysis of radiomic and mRNA data: **(A)** Nephrographic phase; **(B)** Corticomedullary phase; **(C)** Unenhanced phase; **(D)** mRNA data. Based on the clustering results, patients were divided into different clustering groups. The OS and DFS of cases in each group are shown and compared by Kaplan**–**Meier curves. OS, Overall survival; DFS, Disease-free survival.

Using radiomic features of different CT phases and mRNA data, we performed unsupervised hierarchical clustering to present the radiomic profiles of each CT phase and the expressional pattern of mRNA. According to the clustering groups, patients were classified into distinct radiomic or transcriptome subsets (**Figure 2**). Patients’ prognosis was compared within the clustering groups from each CT phase or mRNA data. Notably, clustering from both nephrographic phase and mRNA can divide patients into different prognostic groups (**Figures 2A, D**). Specifically, cases in the clustering groups A and B of the nephrographic phase had statically significant shorter median OS (not reach, 78.4-Mo *vs.* not reach) and DFS (62.8-Mo, 78.4-Mo *vs.* 123.7-Mo) against those in group C, while patients in the clustering groups B and C based on mRNA data harbored significantly poorer prognosis than those in group A (median OS: 74.1-Mo, 118.8-Mo *vs.* not reach; median DFS: 78.4-Mo, 118.8-Mo *vs.* not reach). In contrast, clustering groups from corticomedullary or unenhanced phase shared similar OS and DFS (**Figures 2B, C**). Based on the unsupervised characteristic of the clustering analysis, these findings reflected the strong potential of both nephrographic radiomic features and mRNA data in predicting the clinical outcomes of ccRCC patients, warranting further investigation.

### The Prognostic Value of Radiomics Features and Transcriptome mRNA

**Figure 3:**
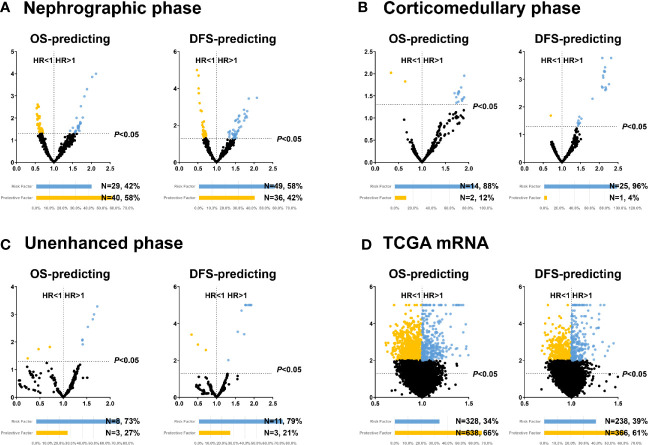
Volcano plots illustrating the univariate COX regression results of features extracted from nephrographic **(A)**, corticomedullary **(B)** and unenhanced **(C)** phase and transcriptome mRNA level **(D)**. OS, Overall survival; DFS, Disease-free survival; HR, Hazard ratio.

We further explored the value of each radiomic feature and mRNA in predicting OS and DFS. The detailed processes of prognostic analyses are shown in **Figures 1B, C**. In univariate analyses, 48, 15 and 10 radiomic features extracted from nephrographic, corticomedullary and unenhanced phase were predictors of both OS and DFS, respectively, while the mRNA FPKM of 521 genes were significantly associated with patients’ prognosis (**Figure 3**, **Table S2**).

**Figure 4:**
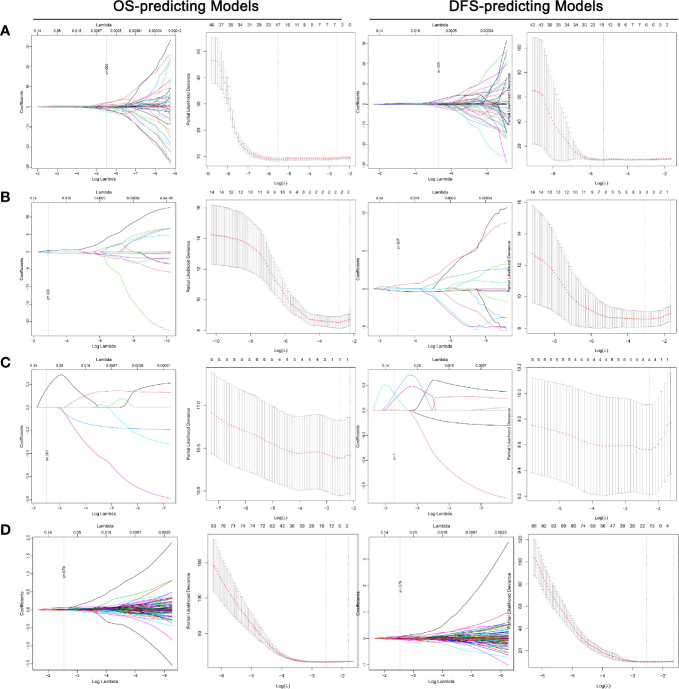
LASSO COX regression for the OS- and DFS-predicting models based on radiomics features extracted from nephrographic **(A)**, corticomedullary **(B)** and unenhanced **(C)** phase and transcriptome mRNA level **(D).** Left plot of each model: The dotted vertical line was plotted at the value selected by the 10-fold cross-validation based on the minimum criteria (the value of lambda with the lowest partial likelihood deviance). Right plot of each model: Selection of the tuning parameter (lambda) in the LASSO regression *via* 10-fold cross-validation based on minimum criteria. LASSO: Least absolute shrinkage and selection operator; OS, Overall survival.

The overall ability of each CT phase and transcriptome mRNA in predicting survival outcomes was further tested. LASSO COX regression was carried out using factors with ability in predicting both OS and DFS in univariate analyses (**Figure 4**). Then, potential OS- and DFS-predictors identified by LASSO COX regression were analyzed by the backward stepwise COX, aiming at generating more exquisite models and eliminating the redundant factors (**Tables S3, S4**). At last, 11 OS-predicting and eight DFS-predicting features were identified in nephrographic phase. Similar number of predictors were confirmed in mRNA data (seven OS-predictors and seven DFS-predictors). On the contrary, very few prognostic features were found in corticomedullary (2 OS-predictor and 2 DFS-predictors) and unenhanced phase (one OS-predictors and one DFS-predictors).

Based on the beta value of predictors included in the backward COX regression, the prognostic models of each CT phase and mRNA were developed (**Tables S3, S4**). The C-index for OS-predicting in nephrographic, corticomedullary, unenhanced phase and transcriptome mRNA model was 0.815, 0.745, 0.680, and 0.856, respectively. In terms of DFS-predicting, the C-index was 0.771, 0.728, 0.643, and 0.823, respectively. Taking together, these findings demonstrated again nephrographic features harbored superior predictive value against features from corticomedullary or unenhanced phases. Besides, transcriptome mRNA data were also capable for prognostic evaluation.

Since radiomic features from the nephrographic phase and mRNA data exhibited strong prognostic power, we further investigated the inner correlation between them (**Figure S1**). The results revealed that a group of GLCM25 radiomic features belonging to the InformationMeasureCorr1 feature set were positively associated with the transcriptional expression of a great number of genes, while another group of GLRLM25 features (LongRunEmphasis and LongRunHigh/LowGrayLevelEmpha feature sets) were negatively associated with the mRNA level of many genes.

The above analyses were carried out based on radiomic features after Combat harmonization. The exact values before and after the Combat harmonization of nephrographic radiomic features included in the final predictive model are shown in **Figure S2**.

### The Development and Validation of the OS- and DFS-Predicting Models Based on Radiotranscriptomics Data

With the hypothesis that the joint usage of the radiotranscriptomics data could further strengthen the performance of the models, we then combined nephrographic features and mRNA data to develop prognostic models. 110 cases with available data on both nephrographic phase and mRNA were included. For each patient, radiomic score and transcriptome score were calculated using nephrographic features and mRNA data (**Tables S3, S4**).

**Figure 5:**
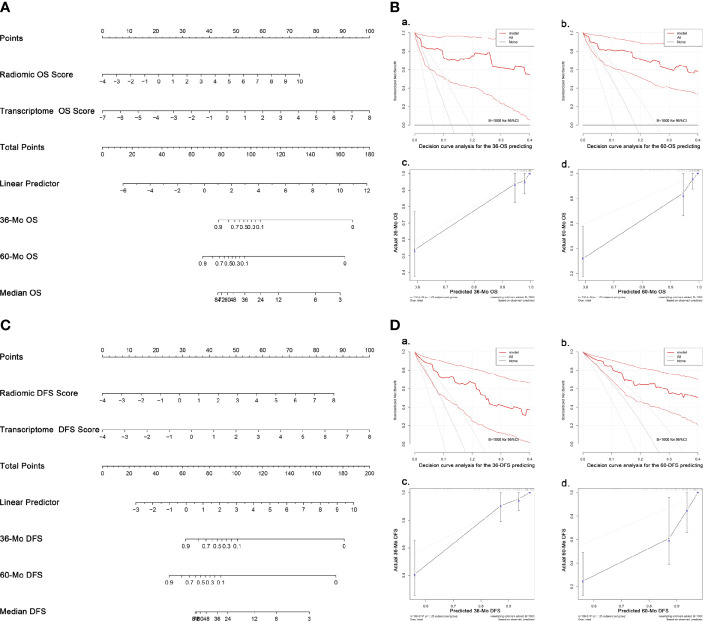
Nomograms and validation of the OS- and DFS-predicting models. **(A)** Nomogram predicting OS. **(B)** Validation of the OS-predicting model: **(A, B)** DCA of the nomogram predicting 36-Mo and 60-Mo OS. **(C, D)** Calibration curves showing the probability of 36-Mo and 60-Mo OS between model prediction and actual observation. **(C)** Nomogram predicting DFS. **(D)** Validation of the DFS-predicting model: **(A, B)** DCA of the nomogram predicting 36-Mo and 60-Mo DFS. **(C, D)** Calibration curves showing the probability of 36-Mo and 60-Mo DFS between model prediction and actual observation. In DCA plot, the X-axis shows the threshold probabilities while the Y-axis shows the net benefit (adding true positives and subtracting false positives). The black horizontal line along the x-axis assumes that no patients died (or progressed) whereas the gray line assumes that death (or progression) occurred in all cases. DCA and calibration curve analyses were carried out using 1000 bootstrapping replications. The bootstrapped 95%CIs of the decision curves and calibration curves are present. OS, Overall survival; DFS, Disease-free survival; DCA, Decision curve analyses, CI, Confidence interval.

In univariate analyses for clinical factors, T stage could predict both OS and DFS, while age was capable of predicting DFS only. In the backward COX regression analysis, only radiomic features and transcriptome data were statistically significant in the OS- and DFS-prediction while no clinical factors were included (**Table 2**). The two models were virtually presented as nomogram (**Figures 5A, C**).

**Table 2:** The development of the OS- and DFS-predicting models.

A. OS-predicting model	Beta value	HR (95%CI)	*P* value	C-index
**Radiomic OS Score**	0.59	1.78 (1.26-2.53)	0.001	0.927
**Transcriptome OS Score**	0.73	2.08 (1.48-2.91)	<0.001	
**B. DFS-predicting model**	**Beta value**	**HR (95%CI)**	***P* value**	**C-index**
**Radiomic DFS Score**	0.58	1.78 (1.25-2.52)	0.001	0.879
**Transcriptome DFS Score**	0.66	1.94 (1.35-2.78)	<0.001	

OS, Overall survival; DFS, Disease-free survival.

The models were then examined using 1000 bootstrapping replications (**Figures 5B, D**). The C-index of the OS- and DFS-predicting model was 0.943 and 0.881, respectively, which were much higher than the radiomics or transcriptome model alone. C-index at different time points also supported that the radiotranscriptomic model had the highest discrimination power (**Figure S3**). Besides, the DCA exhibited great positive net benefits among most of the threshold probabilities, suggesting satisfactory clinical effect of the novel models. Furthermore, calibration curves reflected great consistency between the models predicting survival and actual observation.

## Discussion

The past two decades have witnessed the rapid development of radiomics. In the current study, we firstly investigated the prognostic value of radiomics signatures extracted from CT images in patients with localized ccRCC. According to our findings, radiomics features from nephrographic phase could predict the postoperative prognosis of ccRCC patients with high accuracy, whereas the prognostic value of corticomedullary or unenhanced phases was limited. We further showed that radiotranscriptomics models integrating both radiomics features and transcriptome data along with clinical factors could more precisely predict both OS and DFS.

The application of radiomics analysis could be traced back to the 1970s (22). Nowadays, the application scope of radiomics analysis has extended dramatically and penetrated into various fields of different diseases (3–6). The biggest advantage of radiomics analysis is that, unlike interpreting medical images using human eyes, radiomics provides objective and quantifiable imaging information that could reflect the biological process of different diseases (23). Because of this, radiomics is now recognized as an important biomarker.

Many studies had explored the association between radiomics analyses and clinical outcomes in patients with various cancers (7–9). In RCC, radiomics features were proved to be capable of not only differentiating benign and malignant masses but also predicting the stage and Fuhrman nuclear grade of tumor, two critical prognosticators of ccRCC (5, 10–12). Thus, presumably, radiomics signatures should also be related to patients’ prognosis. Actually, in 2016, there was a study exploring the clinical value of CT textural analysis in large primary RCC which also reported the relationship between radiomics features and clinical outcomes (24). However, a major flaw of that study was the mixture of both M0 and M1 cases as well as cases of different histologic types. Since the standard treatment schemes for these patients differed, it’s improper to evaluate their prognosis using a unified approach.

In this study, we found that nephrographic phase was the best CT phase for prognostic assessment, which was reasonable because nephrographic phase was commonly regarded as the most sensitive phase for tumoral detection (25, 26). Likewise, other studies focusing on the value of radiomics in predicting Fuhrman grade of ccRCC also revealed that models based on nephrographic phase had the highest discrimination power and contained more radiomics features than models based on other phases (10–12). Yet, several researchers reported that the unenhanced phase had better performance in differentiating RCC and angiomyolipoma than other phases (27, 28), suggesting that radiomics features from distinct CT phases might have different advantages in distinct areas.

It was reported that the combination of multi-omics data could strengthen the predictive precision of prognostic models (9). In our study, we found the predictive accuracy of the models increased remarkably when combining both radiomics and transcriptome data. This could be explained by the hypothesis that multi-omics models could reflect the biological characteristics with higher dimension, and thus, more precisely predict tumor progression.

Apart from prognostic assessment for localized ccRCC cases, radiomics was also capable of evaluating the clinical outcomes for advanced ccRCC patients. Early in 2011, Goh et al. reported that CT texture could predict the DFS of tyrosine kinase inhibitors (TKIs) treatment in patients with M1 ccRCC (29). The role of radiomics in predicting TKIs efficacy was also found in gastrointestinal stromal tumors and lung cancers (7, 8). Taking together, our study as well as the previous ones verified that the prognostic significance of radiomics features could be applied in both early and late stage of ccRCC. On the other hand, with the emerging of immunotherapy in advanced RCC (30), future studies are still needed to elucidate the role of radiomics in predicting treatment outcomes of immunotherapy in ccRCC patents.

Several limitations existed in this study. Firstly, this is a retrospective study with shortcomings connected to its retrospective nature. Secondly, the occurrence of outcome events (death and progression) was relatively low, which might hinder the accuracy of prognostic assessment. Thirdly, in our study, radiomics features were extracted from two-dimensional ROI rather than from three-dimensional ROI. Finally, only cases from the TCGA-KIRC were included and only a bootstrapping validation was used in this study, thus, the findings of this study require further external validation using data from other centers. Besides, we will also construct our own cohort in the future to further validate our findings.

## Conclusion

To our knowledge, in this study, we firstly explored the value of radiomics signatures extracted from different CT phases in predicting the survival outcomes of ccRCC patients after nephrectomy. Our findings revealed that features obtained from nephrographic phase harbored promising prognostic ability in predicting both OS and DFS, while the prognostic value of features from corticomedullary or unenhanced phase was relatively weak. Besides, radiotranscriptomics models combining both radiomics and mRNA data exhibited improved predictive accuracy in prognostic evaluation. Our works will facilitate clinicians in better assessing the prognosis of ccRCC patients, and thus, making personalized therapeutic decision.

## Data Availability Statement

The datasets presented in this study can be found in online repositories. The names of the repository/repositories and accession number(s) can be found in the article/**Supplementary Material**.

## Ethics Statement

Ethical review and approval were not required for the study on human participants in accordance with the local legislation and institutional requirements. Written informed consent for participation was not required for this study in accordance with the national legislation and the institutional requirements.

## Author Contributions

XT and Y-lG: conception and design. XT, TP, W-fY, W-lQ, and Z-gY: data collection and analysis. XT: writing the manuscript. Y-lG and Z-gY: revising and editing the manuscript. All authors contributed to the article and approved the submitted version.

## Conflict of Interest

The authors declare that the research was conducted in the absence of any commercial or financial relationships that could be construed as a potential conflict of interest.

## Supplementary Material

Supplementary Figure 1 The inner correlation between radiomic features from the nephrographic phase and mRNA data. Nephrographic radiomic features and genes with p<0.05 and p<0.005 respectively in predicting both OS and DFS were included.

Supplementary Figure 2 The exact values before and after the Combat harmonization of nephrographic radiomic features that are included in the final predictive model.

Supplementary Figure 3 C-index examination of different models predicting either OS or DFS at different time points. 1000 bootstrapping replications were used. OS, Overall survival; DFS, Disease-free survival; C-index, concordance index.
