# Supplementary figures and images for: The Prognostic Value of Radiomics Features Extracted From Computed Tomography in Patients With Localized Clear Cell Renal Cell Carcinoma After Nephrectomy
Source: Front Oncol. 2021 Mar 5;11:591502. doi: 10.3389/fonc.2021.591502 (PMC7973240; doi:10.3389/fonc.2021.591502)

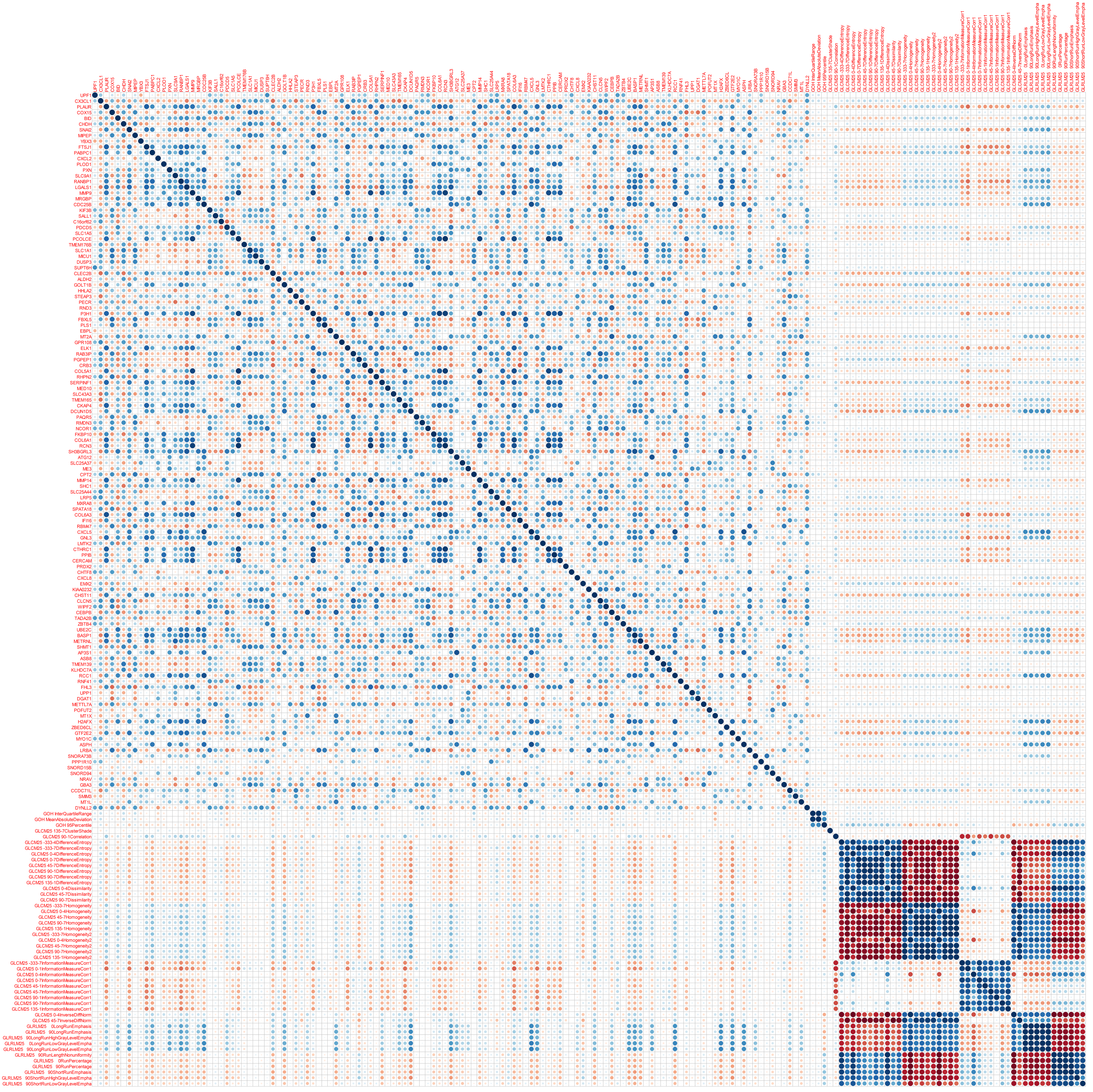

Supplement: Supplementary Figure 1 — The inner correlation between radiomic features from the nephrographic phase and mRNA data. Nephrographic radiomic features and genes with p<0.05 and p<0.005 respectively in predicting both OS and DFS were included. [file Image_1.tif]

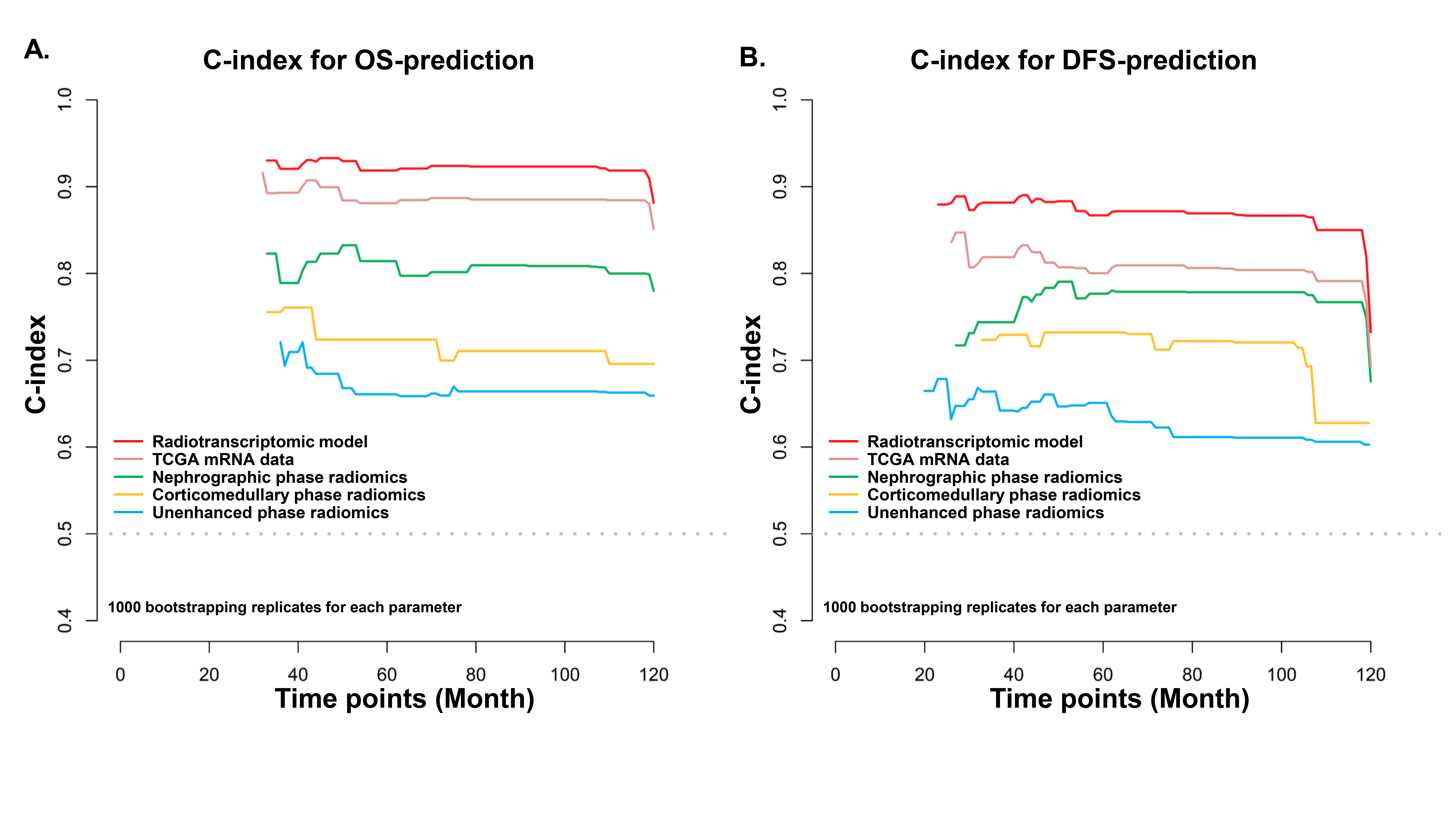

Supplement: Supplementary Figure 3 — C-index examination of different models predicting either OS or DFS at different time points. 1000 bootstrapping replications were used. OS, Overall survival; DFS, Disease-free survival; C-index, concordance index. [file Image_3.tif]
