# Supplementary material for: The Prognostic Value of Radiomics Features Extracted From Computed Tomography in Patients With Localized Clear Cell Renal Cell Carcinoma After Nephrectomy
Source: Front Oncol. 2021 Mar 5;11:591502. doi: 10.3389/fonc.2021.591502 (PMC7973240; doi:10.3389/fonc.2021.591502)
Supplement: Supplementary file 4 [file DataSheet_1.docx]

Supplementary Tables

# Supplementary Tables S1, S3, S4

**Table S1. Radiomics modeling details according to the Imaging Biomarker Standardization Initiative (IBSI) guidelines.**

| **General information** |  |
| --- | --- |
| **Imaging data** | CT |
| **Image acquisition** | The imaging data were collected from seven institutions and three different manufactures: General Electric (GE), Siemens and Philips Medical Systems. The acquisition parameters of CT were as follows: slice thickness, 1 - 5mm; tube voltage, 120 -140 kV; tube current, 160 - 618 mA; display field of view, 278 - 628; matrix, 512 × 512; and pixel size, 0.542 × 0.542 mm2 to 0.976 × 0.976 mm2. |
| **Approach** | 2D |
| **Process workflow** | See Figure 1 |
| **Software** | IBEX |
| **Data availability** | Image data: the TCIA datasets (http://www.cancerimagingarchive.net/)  Software: IBEX (β1.0, http://bit.ly/IBEX_MDAnderson) |
| **Data Conversion** |  |
| **Procedure** | Z-score normalization |
| **Image Post-Acquisition Processing** |  |
| **Procedure** | Not applicable |
| **Segmentation** |  |
| **Tumor site** | Renal tumors |
| **Radiologists** | Two radiologists independently segmented |
| **Delineation method** | Manual delineation |
| **Interpolation** |  |
| **Resampled voxel spacing (mm)** | 1 × 1 (axial) |
| **Image interpolation method** | The cubic B-spline interpolation |
| **Re-segmentation** |  |
| **ROI mask criteria** | Not applicable |
| **Discretization** |  |
| **Discretization methods** | Fixed Bin Size (FBS) |
| **Discretization parameters** | FBS: bin widths = 25 HU |

**Table S3. Multivariate COX regression for OS-predicting using backward elimination strategy**

| **Ⅰ. Nephrographic phase** |  |  |  |  |  |
| --- | --- | --- | --- | --- | --- |
| **Radiomic feature category** | **Radiomic feature** | **Beta** | **HR (95%CI)** | ***P* value** | **C-index** |
| GradientOrientHistogram | InterQuartileRange | 0.48 | 1.61 (0.92-2.82) | 0.093 | 0.815 |
| GrayLevelCooccurenceMatrix25 | 135-7ClusterShade | 0.41 | 1.51 (1.02-2.23) | 0.038 |  |
| GrayLevelCooccurenceMatrix25 | 90-1Correlation | 0.85 | 2.35 (1.02-5.38) | 0.044 |  |
| GrayLevelCooccurenceMatrix25 | 0-7DifferenceEntropy | 2.32 | 10.16 (1.27-81.31) | 0.029 |  |
| GrayLevelCooccurenceMatrix25 | 90-1DifferenceEntropy | -3.51 | 0.03 (0-0.26) | 0.001 |  |
| GrayLevelCooccurenceMatrix25 | 0-1InformationMeasureCorr1 | 2.39 | 10.94 (2.42-49.58) | 0.002 |  |
| GrayLevelCooccurenceMatrix25 | 0-7InformationMeasureCorr1 | -2.97 | 0.05 (0.01-0.33) | 0.002 |  |
| GrayLevelCooccurenceMatrix25 | 0-4InverseDiffNorm | -1.95 | 0.14 (0.03-0.68) | 0.014 |  |
| GrayLevelRunLengthMatrix25 | 0LongRunEmphasis | -4.16 | 0.02 (0-0.27) | 0.004 |  |
| GrayLevelRunLengthMatrix25 | 90LongRunLowGrayLevelEmpha | 4.56 | 95.18 (5.52-1640.39) | 0.002 |  |
| GrayLevelRunLengthMatrix25 | 90ShortRunLowGrayLevelEmpha | -1.63 | 0.2 (0.05-0.72) | 0.014 |  |
| **Ⅱ. Corticomedullary phase** |  |  |  |  |  |
| **Radiomic feature category** | **Radiomic feature** | **Beta** | **HR (95%CI)** | ***P* value** | **C-index** |
| GrayLevelCooccurenceMatrix25 | 135-1ClusterShade | 0.65 | 1.91 (1.16-3.16) | 0.011 | 0.745 |
| Shape | Mass | -0.48 | 0.62 (0.42-0.91) | 0.014 |  |
| **Ⅲ. Unenhanced phase** |  |  |  |  |  |
| **Radiomic feature category** | **Radiomic feature** | **Beta** | **HR (95%CI)** | ***P* value** | **C-index** |
| Shape | MeanBreadth | 0.47 | 1.6 (1.18-2.16) | 0.003 | 0.680 |
| **Ⅳ. TCGA mRNA data** |  |  |  |  |  |
|  |  | **Beta** | **HR (95%CI)** | ***P* value** | **C-index** |
|  | **CD82** | 0.07 | 1.07 (1.04-1.11) | <0.001 | 0.856 |
|  | **ARMC7** | -0.49 | 0.62 (0.48-0.79) | <0.001 |  |
|  | **MED10** | 0.07 | 1.07 (1.02-1.13) | 0.010 |  |
|  | **SLC25A37** | 0.11 | 1.12 (1.05-1.19) | 0.001 |  |
|  | **ME3** | 0.13 | 1.14 (1.04-1.24) | 0.003 |  |
|  | **LRPAP1** | -0.14 | 0.87 (0.79-0.96) | 0.006 |  |
|  | **INAFM2** | 0.08 | 1.08 (1.03-1.13) | 0.003 |  |

**OS: Overall survival; HR: Hazard ratio**

**Radiomic score and transcriptome score were calculated using nephrographic features and mRNA data.**

**For each case, the radiomic and transcriptome score could be calculated using the following formula: x1b1+x2b2+ x3b3+…xkbk, where xk and bk represents the nephrographic feature or mRNA FPKM value and their related beta value, respectively.**

**Table S4. Multivariate COX regression for DFS-predicting using backward elimination strategy**

| **Ⅰ. Nephrographic phase** |  |  |  |  |  |
| --- | --- | --- | --- | --- | --- |
| **Radiomic feature category** | **Radiomic feature** | **Beta** | **HR (95%CI)** | ***P* value** | **C-index** |
| GrayLevelCooccurenceMatrix25 | 135-7ClusterShade | 0.56 | 1.74 (1.21-2.52) | 0.003 | 0.771 |
| GrayLevelCooccurenceMatrix25 | 0-7DifferenceEntropy | 2.33 | 10.28 (2.16-48.99) | 0.003 |  |
| GrayLevelCooccurenceMatrix25 | 45-7Dissimilarity | -2.70 | 0.07 (0.01-0.36) | 0.002 |  |
| GrayLevelCooccurenceMatrix25 | 0-4Homogeneity | -1.40 | 0.25 (0.06-0.97) | 0.045 |  |
| GrayLevelCooccurenceMatrix25 | 0-7InformationMeasureCorr1 | -0.80 | 0.45 (0.3-0.67) | <0.001 |  |
| GrayLevelRunLengthMatrix25 | 0LongRunEmphasis | -2.58 | 0.08 (0.02-0.39) | 0.002 |  |
| GrayLevelRunLengthMatrix25 | 90LongRunLowGrayLevelEmpha | 3.38 | 29.23 (5.11-167.29) | <0.001 |  |
| GrayLevelRunLengthMatrix25 | 90ShortRunLowGrayLevelEmpha | -1.08 | 0.34 (0.13-0.91) | 0.032 |  |
| **Ⅱ. Corticomedullary phase** |  |  |  |  |  |
| **Radiomic feature category** | **Radiomic feature** | **Beta** | **HR (95%CI)** | ***P* value** | **C-index** |
| GrayLevelCooccurenceMatrix25 | 135-1ClusterShade | 0.85 | 2.33 (1.51-3.59) | <0.001 | 0.728 |
| Shape | Mass | -0.40 | 0.67 (0.48-0.92) | 0.015 |  |
| **Ⅲ. Unenhanced phase** |  |  |  |  |  |
| **Radiomic feature category** | **Radiomic feature** | **Beta** | **HR (95%CI)** | ***P* value** | **C-index** |
| Shape | Compactness1 | 0.62 | 1.86 (1.43-2.41) | <0.001 | 0.643 |
| **Ⅳ. TCGA mRNA data** |  |  |  |  |  |
|  |  | **Beta** | **HR (95%CI)** | ***P* value** | **C-index** |
|  | **SNAI2** | 0.10 | 1.10 (1.05-1.16) | <0.001 | 0.823 |
|  | **SLC12A4** | -0.08 | 0.92 (0.84-1.01) | 0.084 |  |
|  | **ARMC7** | -0.16 | 0.85 (0.71-1.02) | 0.073 |  |
|  | **SLC25A37** | 0.13 | 1.14 (1.07-1.21) | <0.001 |  |
|  | **FRG1BP** | -0.20 | 0.82 (0.68-0.98) | 0.031 |  |
|  | **BACE2** | 0.07 | 1.07 (1.03-1.11) | 0.001 |  |
|  | **SNORD15B** | 0.40 | 1.49 (1.01-2.21) | 0.046 |  |

**DFS: Disease-free survival; HR: Hazard ratio**

**Radiomic score and transcriptome score were calculated using nephrographic features and mRNA data.**

**For each case, the radiomic and transcriptome score could be calculated using the following formula: x1b1+x2b2+ x3b3+…xkbk, where xk and bk represents the nephrographic feature or mRNA FPKM value and their related beta value, respectively.**
